# Research on the Sensing Performance of the Tuning Fork-Probe as a Micro Interaction Sensor

**DOI:** 10.3390/s150924530

**Published:** 2015-09-23

**Authors:** Fengli Gao, Xide Li

**Affiliations:** 1 Department of Engineering Mechanics, AML, Tsinghua University, Beijing 100084, China; E-Mail: gaofengli@gmail.com; 2 Center for Nano and Micro Mechanics, Tsinghua University, Beijing 100084, China

**Keywords:** tuning fork, scanning near-field optical microscopy, finite element method, force or interaction sensing harmonic response

## Abstract

The shear force position system has been widely used in scanning near-field optical microscopy (SNOM) and recently extended into the force sensing area. The dynamic properties of a tuning fork (TF), the core component of this system, directly determine the sensing performance of the shear positioning system. Here, we combine experimental results and finite element method (FEM) analysis to investigate the dynamic behavior of the TF probe assembled structure (TF-probe). Results from experiments under varying atmospheric pressures illustrate that the oscillation amplitude of the TF-probe is linearly related to the quality factor, suggesting that decreasing the pressure will dramatically increase the quality factor. The results from FEM analysis reveal the influences of various parameters on the resonant performance of the TF-probe. We compared numerical results of the frequency spectrum with the experimental data collected by our recently developed laser Doppler vibrometer system. Then, we investigated the parameters affecting spatial resolution of the SNOM and the dynamic response of the TF-probe under longitudinal and transverse interactions. It is found that the interactions in transverse direction is much more sensitive than that in the longitudinal direction. Finally, the TF-probe was used to measure the friction coefficient of a silica–silica interface.

## 1. Introduction

Scanning near-field optical microscopy (SNOM) was developed more than three decades ago [1,2]. Because of its capacity to break through the boundaries of conventional diffraction-limited optical microscopy and no-contact sensing property, the applications of SNOM have been extended to near-field spectroscopy [3,4,5,6,7], topographic detecting [8], residual stress measurement [9], and soft matter testing [10,11,12]. Generally, a commercial scanning near-field optical microscope employs a tapered optical fiber as a probe to obtain near-field optical signals as well as topographic images. The high-resolution imaging of SNOM is achieved when the probe-sample separation is precisely controlled in the near-field regime. Therefore, various distance regulation schemes have been proposed, such as electron tunneling [2], frustrated total internal reflection [13], background fluorescence increases [14], and the shear-force control schemes [15,16], which are now widely used owing to their simplicity, compactness, reduced drift in the feedback loop, and lower levels of the parasitic optical background [17]. 

The shear-force detection technique uses a tuning fork (TF) to produce probe oscillation parallel to the sample surface and measure its oscillation amplitude [18,19,20,21], where a tapered optical fiber is attached to one prong of the TF, and the whole structure works at its resonance under a harmonic voltage excitation. Normally, as the probe approaches the sample surface, the oscillation amplitude of the probe decreases, and sometimes its resonance frequency is shifted to a higher frequency. Therefore, investigating the dynamic property and understanding the capability of controlling the probe–sample distance of this TF-probe are critical issues to improve the image quality of SNOM or the sensitivity of the TF-probe sensor when applied to the measurement of displacement or of interactions between the probe tip and the sample surfaces.

In previous studies, researchers adopted the single degree of freedom vibration theory or the Euler–Bernoulli beam theory to analyze the dynamic behavior of the TF-probe [17,22,23], and concluded that the shear force mechanism was induced by the lateral interaction between the probe and the sample. Meanwhile, various experiments have also been implemented to investigate the shear force mechanism of the TF-probe. For example, beam diffraction techniques were used to measure the actual oscillation amplitude of the probe vibration [24,25]. Thereafter, Gregor *et al.* [26] studied in detail the probe–surface interaction by measuring dither resonance profiles and approach curves in a vacuum and in liquid helium. They concluded that the so-called shear-force mechanism was a direct, short-range, mechanical contact between the probe and the sample surface. However, when studying the shear force between a glass microprobe and a mica surface under controlled humidity, Okajima and Hirotsu [20] found that direct contact was not the only mechanism responsible for the shear force between the tip and surface. Obviously, the influence of environmental conditions and the interaction vicinity of the sample surface have significant influences on the dynamic behavior of the TF-probe.

This finding has led to further in-depth research. Shelimov *et al.* [17] studied the factors leading to a decrease in the resonance quality of TF-probe using a simple elasto-mechanical analysis method. Recently, based on the non-linear tension-bending coupled vibration theory, we established dynamic equations of the shear force system when the TF prong and the attached fiber probe were all elastic deformable structures [27]. The amplitude–distance curves (approaching curves) and amplitude–frequency response curves were obtained, and the impacts of the simplified solutions of the previous research on the properties of the probe approach and its amplitude–frequency responses were discussed given a Van der Waals interaction between the probe tip and the sample surface. Meanwhile, the viscous resistance of a liquid film on the surface of a single crystal silicon wafer was also investigated using the linear beam-bending vibration theory.

Several studies proposed and tested strategies for recovering a high quality (*Q*) factor. Yoo *et al.* [28] showed that the asymmetric frequency response of the TF-probe could be used to increase *Q* factors and suppress the background feedback signal. Moreover, the influences of environmental conditions on shear-force distance control were also investigated. The capillary force caused by the presence of the thin water adhesion layer at the surface was shown to be the main dissipation factor for SNOM measurements in ambient conditions [29,30,31]. The electrostatic force was found to be the most influential factor on the shear-force of the TF-probe and be independent from the nature of the probe tip or the sample [32]. As the tip-to-sample distance decreases, other forces are involved and cause interactions that depend on the chemical nature of the tip and sample surfaces. Research into these areas has led to the development of diverse shear-force distance control sensors over the last decade [33,34,35].

Theoretical and experimental studies have revealed a variety of dynamic performances by the TF-probe. The individual impacts by various factors, such as the dimension, density, the Young’s modulus of the glued probe, the temperature and humidity of the experimental environment, and the interaction between the probe and the sample surface, are difficult to separate out with theoretical analysis or experimental measurement. Therefore, some numerical methods have also been employed to analyze the dynamic performance of the TF-probe. For example, Schmidt *et al.* [22] initiated a finite element method (FEM) to model a complete TF setup and estimated the damping force between a fiber apex and the hydrophilic samples. Additionally, Lee *et al.* [36] analyzed the resonance frequency of quartz TF crystal with FEM and fabricated a TF using photolithography. They compared the discrepancy between the modeled and experimentally measured resonance frequencies. Friedt *et al.* [37] compared the results of experimental tests and FEM modeling of the tip-loaded quartz TF oscillation amplitude, and they demonstrated that the oscillation amplitude might become a limiting factor of the lateral resolution of a shear force microscope.

In addition to the studies on the dynamic behavior of TF-probe, recently some researchers have extended the application of TF-probe as a force sensor used in nanotribological studies [38], nanomechanical characterization [39,40], and as a mass sensor used in electron microscopes to track oscillation amplitude visually [41].

Although these studies have improved the understanding of dynamic behaviors of the TF-probe, the effects of the structural parameters on the sensing performance of TF-probe is still not very clear. Especially, when the TF-probe is used as a force or mass sensor, the relation between the sensitivity and the various structural parameters of the TF-probe needs to be further studied. The goal of this paper is to analyze the influences of multi-parameter on the sensitivity of TF-probe, and to investigate the impact factors on the force sensing of TF-probe. The research involves the combination of experimental results and FEM analysis to determine the dynamic behavior of the TF-probe and presents clear experimental evidence for the effect of atmosphere on the TF-probe *Q* factor. The calculated results reveal the influence of various parameters on the resonance performance of the TF-probe, including the dimensions of the fiber probe, the Young’s modulus of the TF, and the fiber and glue properties, as well as the damping of the interaction (or the environmental damping). To verify the consistency of FEM modeling, the calculated frequency spectrum of the TF-probe was compared with the experimental data provided by our recently developed, single-point laser Doppler vibrometer (LDV) system [27,42]. This system can measure the probe oscillation amplitude with high displacement sensitivity (resolution 50 pm). Then, the calculated data were fit to the experimental resonance spectrum to finely tune the elastic parameters of the probe and glue. The damping constant of the TF-probe was determined by combining the measured *Q* factors. We also investigated the parameters affecting the spatial resolution of SNOM and the dynamics of the TF-probe under Van der Waals interaction and viscous force due to wetting of the probe apex. The oscillation amplitude data obtained with our FEM calculation and beam vibration theory were compared, and the shear interactions in both the longitudinal and lateral directions, using the TF-probe as a force sensor, were calculated by coupling the experimentally acquired frequency spectra. Our results indicate that the single-point LDV method is more sensitive and convenient than other optical methods, such as optical diffraction, to employ in TF-probe oscillation amplitude measurements for using a shear force mechanism to sense forces.

## 2. Resonance Properties Measurement

### 2.1. Geometric and Material Parameters of the TF

**Figure 1:**
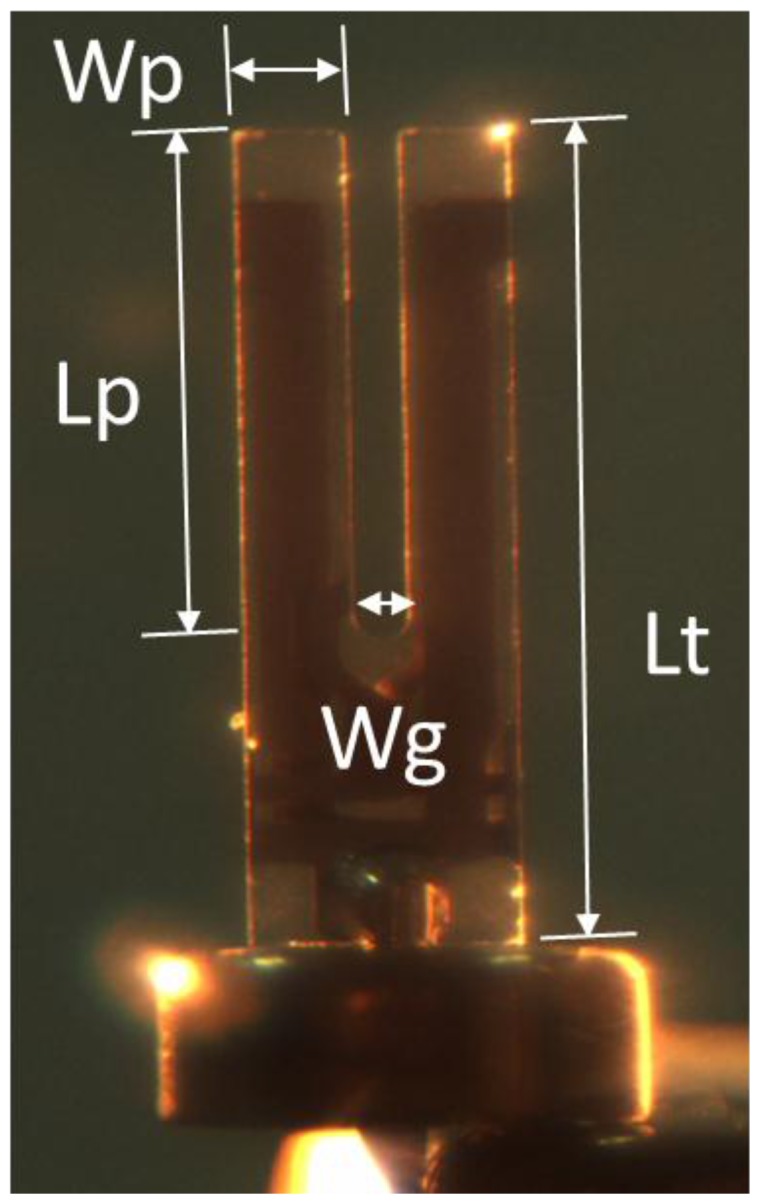
The geometry of a TF.

A TF (Figure 1) is the key component of a shear-force sensor. In our experiments, commercial TFs were used with a nominal resonance frequency of 32.768 kHz. Their geometric parameters were measured by an optical microscope. The geometric dimension variance values were introduced by both their manufacturing and image processing. To minimize this deviation, the mean dimensional values of nine random selected TFs were measured (Table 1). In addition to these size parameters, other geometric parameters were also measured, such as the face and side electrodes. All these geometric parameters were used in the FEM analysis.

**Table 1:** Geometric dimensions measurements of nine random tuning forks (TFs).

No.	Total Length *L*_t_ (mm)	Prong Length *L*_p_ (mm)	Prong Width *W*_p_ (mm)	Prong Height *H*_p_ (mm)	Gap Width *W*_g_ (mm)
1	6.104	3.887	0.618	0.355	0.303
2	6.026	3.781	0.592	0.339	0.299
3	6.037	3.793	0.592	0.334	0.281
4	6.017	3.743	0.617	0.337	0.289
5	5.983	3.760	0.586	0.342	0.297
6	6.077	3.778	0.621	0.342	0.299
7	6.005	3.739	0.593	0.358	0.298
8	6.017	3.744	0.604	0.348	0.299
9	5.991	3.752	0.615	0.350	0.282
Mean	6.037	3.787	0.608	0.359	0.297

One important dynamic TF characteristic is its resonance frequency, which is determined by both its mass and stiffness as well as by its boundary condition, geometry, density, and Young’s modulus. Table 2 provides the values of these material parameters for a tungsten probe that is glued to the TF prong with epoxy resin.

**Table 2:** Material parameters of a tungsten probe glued to a TF prong with epoxy resin.

	Silica	Chromium	Tungsten	Epoxy Resin [43,44]
Density (kg/m^3^)	2650	7190	1925	2
Young’s modulus (GPa)	78	279	411	2–20
Shear modulus (GPa)	33	115	161	-
Damping coefficient (Ns/m)	7 × 10^−6^	7 × 10^−6^	0.005	-

### 2.2. Frequency Spectrum of a TF

**Figure 2:**
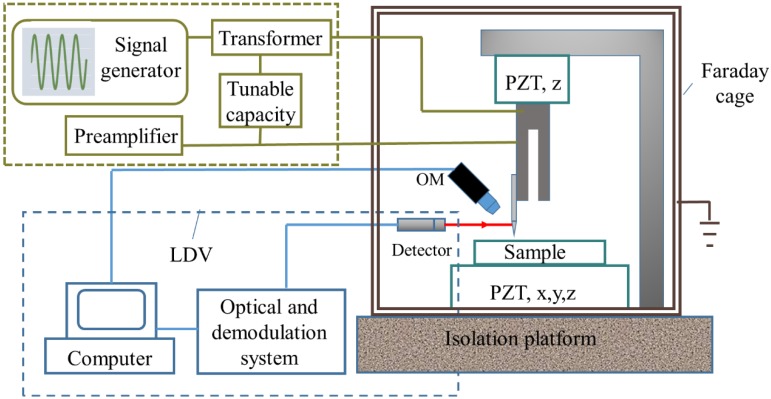
Setup of the frequency spectrum measurement system using an LDV and an electrical feedback mode.

We used a signal generator (AFG-2225) to excite the TF in sweep frequency mode and synchronously recorded the oscillation amplitude of the TF prong with a LDV. A schematic of this setup is shown in Figure 2. The shifted frequency of the laser beam is proportional to the velocity of the moving object owing to the Doppler effect. The displacement resolution of this device is 50 pm. An alternative method named electrical feedback is also presented in Figure 2 to be used in the constrained environment where the LDV system cannot be used. The electrical feedback mode can provide the dynamic response of the TF-probe caused by piezoelectricity. This method is used to measure the *Q* factor under various pressures in Section 2.3.

**Figure 3:**
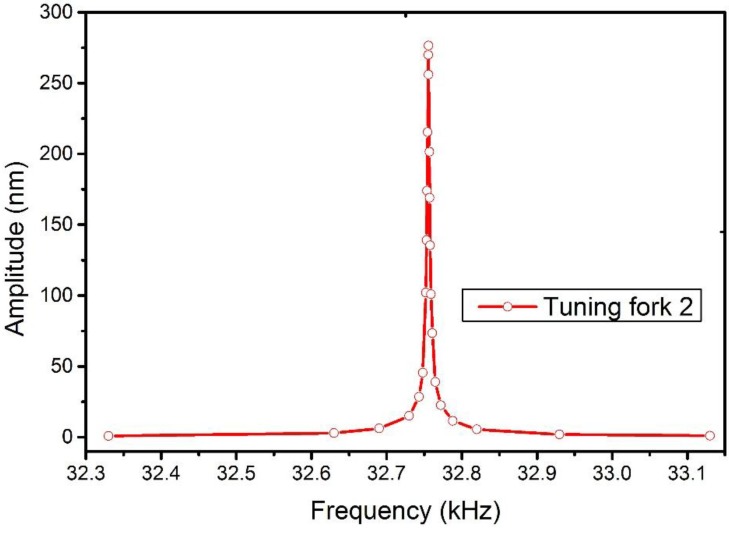
A typical frequency spectrum of a TF.

**Figure 4:**
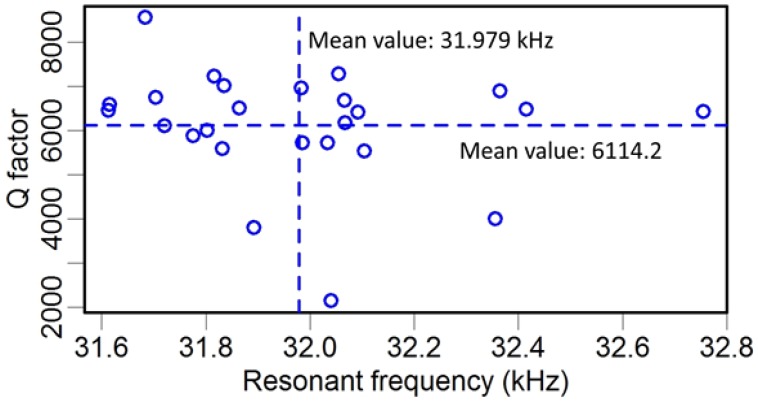
Resonance frequency and *Q* factor of TF.

There is much more advantage to applying a LDV to measure the vibration signal compared with the method used in SNOM (Electrical feedback mode in Figure 2). An LDV is capable of giving the displacement oscillation amplitude of a vibrating object. This is important when a TF is used as a force sensor. While in a SNOM system, an electrical circuit is set to collect the voltage signal induced by the piezoelectricity of the vibrated TF-probe. One problem with this setup is that it cannot directly obtain the displacement of the TF and, thus, a calibration procedure is needed to determine the relationship between the TF oscillation amplitude and the collected voltage signal. The other problem is that the resonance of the TF is coupled with other components in the circuit, especially the capacity of the TF. To eliminate this factor, a tunable capacity was connected in parallel to the TF. Though a good resonance shape can be found after subtly tuning the capacity, there is no quantitative method to evaluate the precision of this operation. Figure 3 shows a typical frequency spectrum of a TF with resonance frequency *f_R_* = 32.760 kHz and quality factor *Q* = 6000 (defined as *f_R_* / Δ*f_FWHM_*, where Δ*f_FWHM_* is the full width of the resonance peak at half maximum). Higher *Q* factors are needed to increase the sensitivity of dynamic sensors. We observed one group of TFs and recorded their resonance frequencies and *Q* factors (Figure 4). Table 3 shows the *Q* factor and resonance frequency recorded before and after gluing a tungsten probe on a TF prong. The length of the fiber is around 2 mm, and its diameter is 80 μm.

**Table 3:** Resonance frequency and *Q* factor of TF-probe.

No.	*f*_R_ of TF(kHz)	*Q* Factor of TF	*f*_R_ of TF-Probe (kHz)	*Q* Factor of TF-Probe
23	32.755	6422	32.472	1353
24	31.684	8563	31.099	2962
25	31.835	7014	31.455	1430

### 2.3. Effect of Air Pressure on the Q Factor

Improving the *Q* factor is critical to developing an effective dynamic force sensor. Since the TF-probe may be used in different pressure environment, the impact of the pressure on Q factor of the TF-probe is investigated in this section. When the tuning fork is vibrating in its resonance frequency, the relationship of drag force-displacement oscillation amplitude with other factors can be written as [45].

(1) $$F_{D0}=i\frac{k_{stat}}{Q}R$$

where *F*_*D*0_, *k_stat_*, and *Q* are the drag force, effective stiffness, and *Q* factor of the TF, respectively. *R* is the oscillation amplitude and *i* is the imaginary unit, indicating that there is a *π* / 2 phase difference between the *F*_*D*0_ and *R*. The resolution of *R* is dependent on the displacement measuring method (the LDV method is used in this study), and there is an inverse relationship between *F*_*D*0_ and *Q*, which means that higher *Q* factor will lead to an improved force resolution.

**Figure 5:**
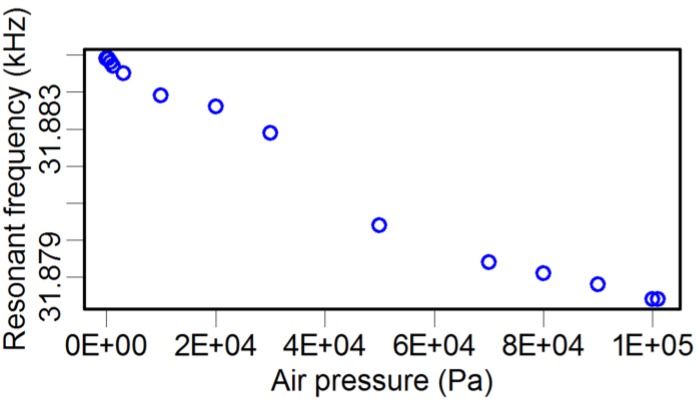
Atmosphere pressure *vs.* the resonance frequency of the TF.

**Figure 6:**
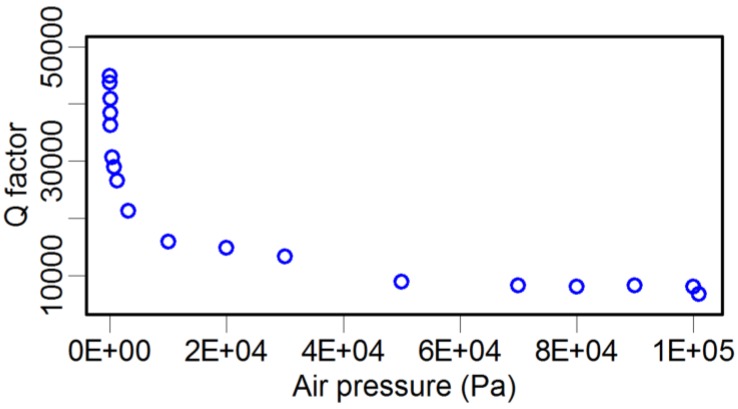
Atmosphere pressure *vs.* the *Q* factor of the TF.

**Figure 7:**
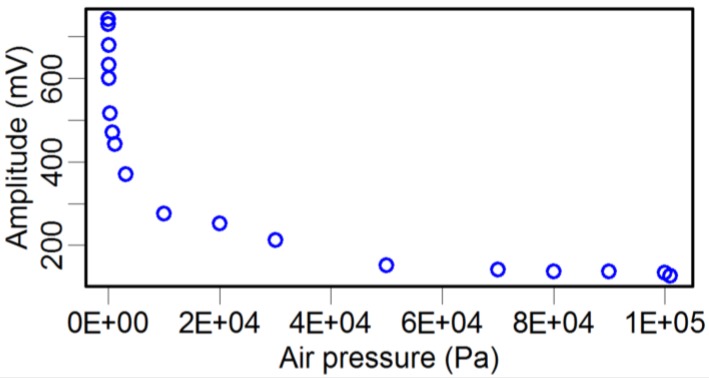
Atmosphere pressure *vs.* the oscillation amplitude of the TF.

As illustrated in Table 3, attaching a probe to the TF dramatically decreases the *Q* factor by three to ten times. In fact, there are many factors affecting the *Q* factor, such as the geometric dimension and shape, material mechanical parameters of both the glue and the probe, and environmental conditions. Here, we investigated the influence of air pressure using an environmental chamber. A set of TFs were laid inside the chamber, and the air pressure was tuned from 3.5 Pa to 101 kPa. Because of the infeasibility of putting the LDV inside the chamber, an alternative method was adopted, where an oscilloscope was used to monitor the induced voltage signals in the TF due to its piezoelectricity. The recorded voltage signal was linearly dependent on the oscillation amplitude of the TF prong. By this method, the resonance frequency, *Q* factor, and oscillation amplitude of the TF were recorded simultaneously, and the results are shown in Figure 5, Figure 6 and Figure 7.

The resonance frequency (Figure 5) was slightly modified by varying the atmosphere pressure, from 31.879 kHz to 31.885 kHz. The lower pressure condition resulted in fewer air molecules adhered to the TF surface than the higher pressure condition, and, hence, the lower pressure condition reduced the mass of the whole vibrating system. However, because the adhered mass of the air molecules is quite small compared with that of the TF, the variation of resonance frequency was not significant. 

In contrast, the effect of atmosphere pressure on the *Q* factor was fundamentally different. The *Q* factor value was nearly eight times larger under the low-pressure condition compared with that in the normal atmosphere. This result strongly indicates that the atmosphere environment dominates the damping behavior of the TF when a TF sensor is used under normal atmospheric conditions. This result is similar to the conclusion of Zhang *et al.* [46], where the “effective” viscous damping was dominant in the high-pressure range.

**Figure 8:**
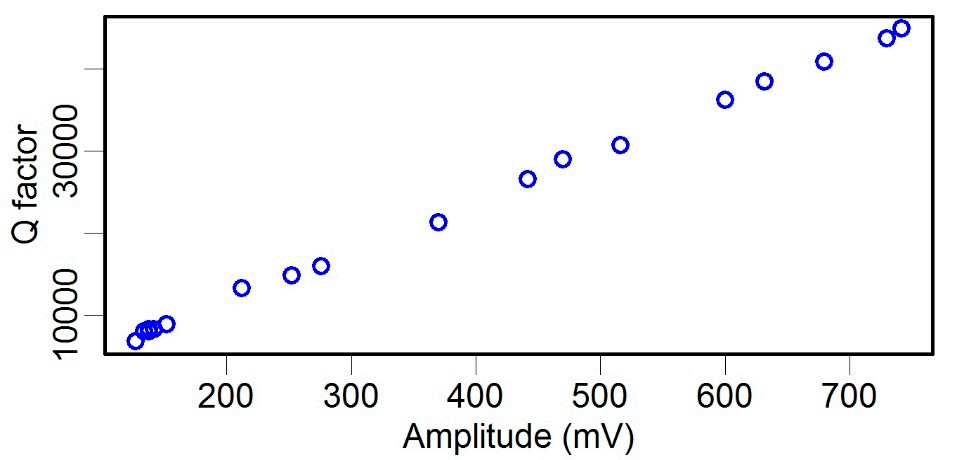
Resonant oscillation amplitude *vs. Q* factor of the TF.

The effect of the atmosphere pressure on the oscillation amplitude (Figure 7) was similar to its effects on the *Q* factor, which implies a linear relationship between the *Q* factor and the oscillation amplitude. According to the linear vibration theory, when the damping effect is small, the *Q* factor has the form:

(2) $$Q=\frac{R\Omega \sqrt{MK}}{F}$$

where *M*, *K*, and *F* are the effective mass, stiffness, and resonant excitation force, respectively, each of which were kept constant during our tests, *R* is the resonant oscillation amplitude of the structure, and Ω is the resonance frequency, which increased slightly when the pressure was deceased (Figure 5). Thus, Equation (2) indicates that the *Q* factor of the system was linearly dependent on the oscillation amplitude, and Figure 8 shows experimental results that are consistent with this theory.

**Figure 9:**
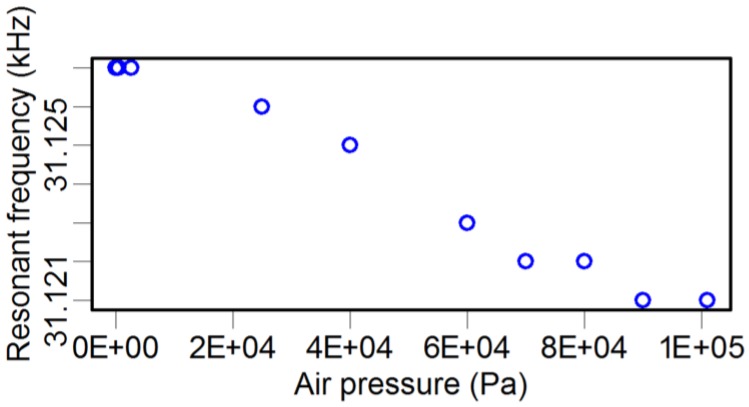
Resonance frequency *vs.* atmospheric pressure of a TF-probe.

**Figure 10:**
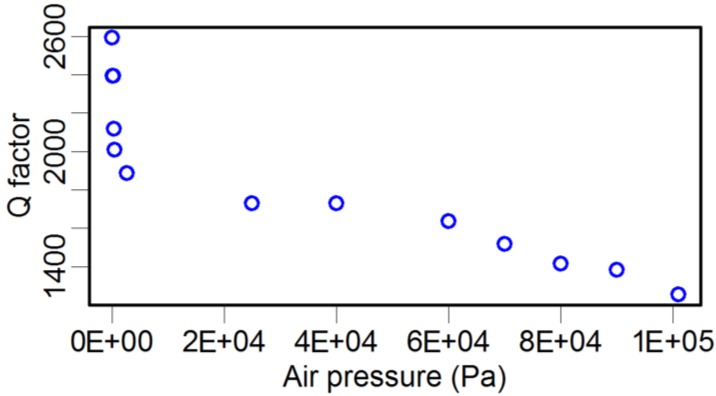
*Q* factor *vs.* atmospheric pressure of a TF-probe.

**Figure 11:**
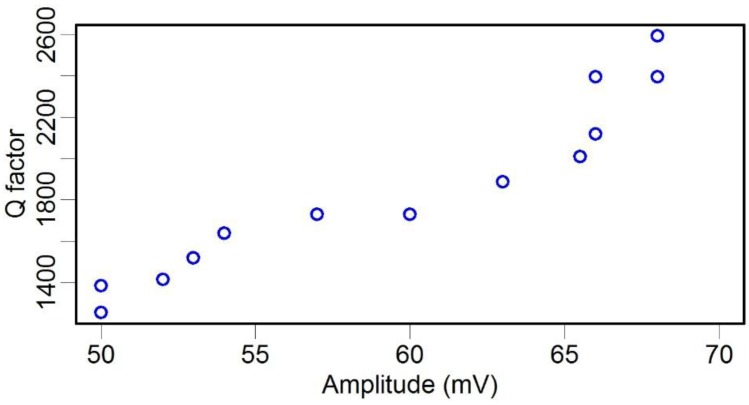
*Q* factor *vs.* the resonant oscillation amplitude of a TF-probe.

For comparison, we also measured the *Q* factor and resonance frequency of the TF-probe under different pressures in the chamber. The resonance frequency before and after gluing a probe to one prong of the TF were 31.542 kHz and 31.121 kHz, respectively. When we increased the pressure, the resonance frequency varied slightly, from 31.121 kHz to 31.127 kHz (Figure 9). The *Q* factor was more sensitive to pressure, changing from 2600 to 1230 as the pressure increased (Figure 10). We examined the relationship between the *Q* factor and the oscillation amplitude and found that they have a roughly linear relationship (Figure 11). The reason of TF-probe shows worse linearity between *Q* factor and air pressure may include two aspects: First, gluing a probe to a prong of TF changes its geometry symmetry, which reduces the *Q* factor and its anti-interference ability. There is local residue deformation during the epoxy curing process. Second, if the material of probe is metal, such as tungsten, the local piezoelectricity will be slightly distorted.

## 3. Dynamic Property Optimization of the TF Sensor and Its Application in Force Detection

### 3.1. Optimization of the Q Factor

**Figure 12:**
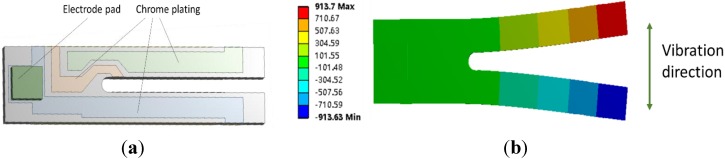
(**a**) The geometry; and (**b**) the fourth mode shape of the TF with FEM analysis.

To optimize the dynamic behavior of the TF-probe, we used FEM to simulate its working state. This analysis focused on the resonant response of the TF-probe; therefore, modal and harmonic analyses were conducted. Because the TF oscillation amplitude is very small, only a few nanometers, we modeled the TF-probe body with a linear elastic solid element. The geometric parameters of the FEM model is listed in Table 1, and its material constants can be found in Table 2. It is important to point out that the electrode pad shown in Figure 12 is composed of two square blocks, while the geometry in practice (Figure 1) is irregular and depends on the welding process. The grids were automatically meshed, and the number of elements was increased if the solution did not converge. The nodes in each of the interfaces, between all contact materials, are bonded. The base facets and the surfaces of the two electrode pads were fixed to simulate the actual boundary condition. In the case of harmonic analysis, the excitation loads were two bending moments, symmetrically located on both end surfaces of the prong. In the analysis of the interactions between the TF-probe and the sample, the interaction force was applied to the probe tip surface (Section 3.2).

The results of a modal analysis show that this structure had a resonance frequency of 32.556 kHz (Figure 12b). This value is close to the nominal value 32.768 kHz, and the mean measured value was 31.979 kHz (Figure 4). In addition to the measurement error, the humidity and the air pressure could also be reasons for this discrepancy. When employing the TF as a dynamic force sensor, a tapered probe glued to one prong could extend the detection ability to a microscopic scale. As the measurement results indicate in Table 3, the glued probe produced two changes: (1) reduction of resonance frequency due to the increase of mass and the change of stiffness; and (2) decrease of *Q* factor due to the increase of damping effect.

Here, we used FEM analysis to focus on the factors that influent the *Q* factor. The material parameters of the TF, epoxy resin and the probe in the calculation are listed in Table 2, and the measured geometric parameters of the TF is listed in Table 1. In the modeling of the TF-probe, we set the middle value of 10 GPa as the Young’s modulus of the epoxy resin (nominal value in the range of 2–20 GPa [43]). Total length and the diameter of the probe were 1.8 mm and 80 μm, respectively, and the radius of the probe tip was 10 μm with a taper angle of 30°. The length, width, and height of the epoxy resin were 0.8 mm, 0.15 mm, and 0.1 mm, respectively. The damping coefficient of glass/epoxy composite in Kabir’s work is in a range of 0.021–0.037 Ns/m [44]. Considering the damping coefficient of glass material is far less than these values, the coefficient value of the epoxy material in our analysis was used as 0.2 Ns/m, and the *Q* factor corresponding to this coefficient in the calculation was in agreement with that of our tests (Figure 10 and Table 4). 

We used harmonic response analysis to determine the unknown damping coefficient of the TF and the probe. The damping coefficient of TF is related to the *Q* factor. The results of the air pressure experiments (Figure 6) for TF shows the *Q* factor closes to 40,000 when air pressure decreased to 3.5 Pa, so we chose *Q* = 40,000 as the target value of *Q* factor in harmonic response analysis for TF in vacuum state. By iterative calculation, we found that the optimal damping coefficient of the TF was 7 × 10^−6^ Ns/m. Another unknown parameter was the damping coefficient of the probe. Adopting the same method as above, based on the *Q* factor of TF-probe in Figure 10, we used *Q* = 4000 as the target value in harmonic response analysis for TF-probe in vacuum state. The calculation results showed the damping coefficient of the probe was 0.005 Ns/m.

**Figure 13:**
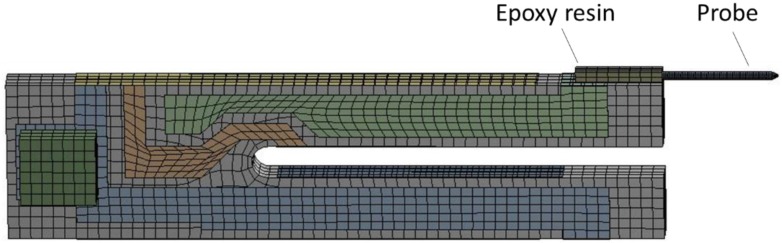
Geometry and meshing of the TF-probe with FEM analysis.

The preset parameters combined with the geometry of TF-probe in Figure 13 is the prototype of all the following FEM simulations. In these calculations, all parameters were kept constant and were the same as the parameters used in the prototype, except for special addressing. These parameters includes density, Young’s modulus, geometric dimensions, and damping coefficient of TF, epoxy, and probe, respectively (refer to Table 1 and Table 2, and Figure 4). Moreover, to simulate a practical SNOM system, all the excitation forces in FEM analysis were assigned to such a value so that the oscillation amplitude of the TF-probe is around 10 nm. 

To optimize the dynamic properties of the TF-probe, we selected low damping materials to increase the *Q* factor. Table 4 illustrates the impact of the damping coefficient of the epoxy resin and the probe independently. Although increasing the damping coefficient of the probe reduced the *Q* factor, it has a limit value of 4182. The strong correlation between the *Q* factor and the damping coefficient of the epoxy resin suggests that using a low damping epoxy resin should be a preferred method. This result also reveals that the energy dissipated by epoxy resin was larger than that of the attached probe.

**Table 4:** The impact of the damping coefficient on the *Q* factor.

Damping Coefficient of the Probe (Ns/m) *	*Q* Factor	Damping Coefficient of the Epoxy Resin (Ns/m) ^#^	*Q* Factor
0.05	3037	0.6	1431
0.005	4025	0.2	4025
0.0005	4182	0.06	10,063
0.00005	4182	0.02	17,889

* Damping coefficient of the epoxy resin = 0.2 Ns/m; ^#^ Damping coefficient of the probe = 0.005 Ns/m.

**Table 5:** The impact of the Young’s modulus on the *Q* factor.

Young’s Modulus of the Probe (GPa) *	*Q* Factor	Young’s Modulus of the Epoxy Resin (GPa) ^#^	*Q* Factor
411	4025	20	2106
200	3926	10	4025
78	3350	6	6311
10	1516	2	13,691

* Young’s modulus of the epoxy resin = 10 GPa; ^#^ Young’s modulus of the probe = 411 GPa.

When using various epoxy resins and probe materials, such as tungsten, steel and glass, the Young’s modulus of the materials are also changeable. Table 5 demonstrates the relationship between the Young’s modulus and the *Q* factor. Values in the first column of Table 5 correspond to the Young’s modulus of materials, tungsten, steel, glass, and epoxy resin, respectively. Reducing the Young’s modulus of the probe will increase its flexibility, hence raising the energy dissipated by the probe deformation. When decreasing the Young’s modulus of the epoxy resin, the elastic connection between the probe and the TF is relaxed; thus, the probe tends to make rigid rotations instead of bending. The damping effect of TF-probe is related to the internal friction of the structure, which has a strong relationship with bending deformation. Thus, a low Young’s modulus of the epoxy resin will lead to a high *Q* factor.

**Figure 14:**
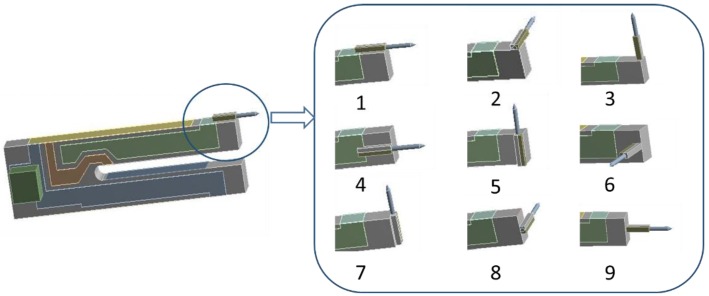
Nine types of TF-probe structures.

**Table 6:** The impact of the gluing forms on the *Q* factor.

Type	*Q* Factor
1	12,913
2	22,253
3	29,306
4	20,821
5	40,337
6	17,922
7	37,934
8	22,241
9	6844

As revealed by the above analysis, the dynamic mechanical properties of various components in the TF, specifically the epoxy resin and the probe, are interrelated with both the deformations and the *Q* factor of the TF-probe. Similarly, gluing a probe on the different surfaces of the TF and inducing varying deformations will cause distinct values of the *Q* factor. Figure 14 displays nine types of TF-probe, and Table 6 illustrates the dependence of the *Q* factor on the gluing forms. In this numerical analysis, the geometry of the probe and the epoxy resin were kept constant, the dimensions of which can be found in Section 2. These results demonstrated that *Q* factors in Types 1, 6, and 9 were relatively small owing to the probe being glued in the worst locations in these types. In Type 1, the gluing surface had the biggest deformation positions of the TF under pure bending vibration. In Types 1, 6, and 9, the stretching of the probe body out of the TF prong also made strong bending movements, which inevitably caused damping effects. Types 3, 5, and 7 are the opposite of Type 1. The gluing surfaces in these types have slight (Type 5) or no (Types 3 and 7) deformations, and the probes made rigid movements without any bending deformations. Types 2, 4, and 8 were in intermediate states. Though each one had some bending movement, the deformations in these gluing positions were relatively small compared with those in Type 1. As illustrated by Table 3 and Figure 4, the values of *Q* factor scatter in a relative large range. In fact, gluing the probe will introduce more factors to affect the *Q* factor, such as the amount and location of the glue. Thus, the comparison in Table 6 is rough.

### 3.2. Dynamic Response of the TF Sensor under Longitudinal and Transverse Interactions

**Figure 15:**
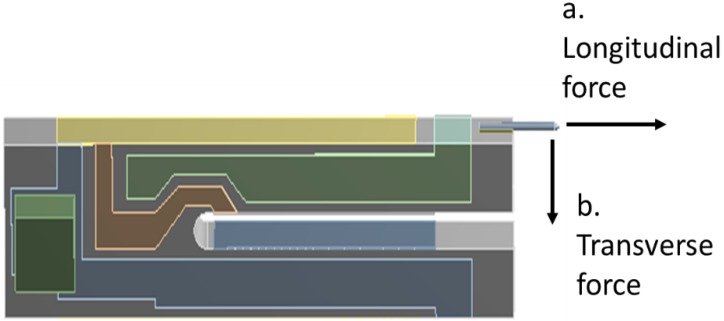
A schematic of the TF-probe under longitudinal and transverse forces.

While our analysis in the last section suggested that choosing a suitable probe gluing form was an effective way to improve the *Q* factor, the most common probe gluing form in SNOM is Type 1 owing to the simplicity of controlling the probe–sample distance with this form and some historical reasons [1,2,8]. Therefore, we used a Type 1 TF-probe to research the dynamic response under two types of loads on the probe tip, the longitudinal force and transverse viscous force (Table 6). First, in a longitudinal interaction analysis, the free oscillation amplitude of the probe tip was set to 10.112 nm (without interaction) and the TF-probe was subjected to a longitudinal interaction (Figure 15a). Table 7 shows the dynamic responses we observed when we changed the longitudinal force. The oscillation amplitude of the probe tip dropped to 4.208 nm when the longitudinal force was increased to 50 mN. This result can be attributed to the growing stiffness of the whole system, which slowly changes the resonance frequency and its oscillation amplitude [27]. Along with these changes, the phase angle between the displacement and the excitation force varied from 91.11° to 155.36°. The dependencies of both the oscillation amplitude and phase angle on the interaction force can be applied to characterize the longitudinal interaction (longitudinal force); however, the phase angle signal is better for this measurement because of its high sensitivity. Another advantage to using the phase angle signal is that a very low excitation voltage can be used, leading to the probe tip having a smaller oscillation amplitude, which makes high spatial resolution achievable.

**Table 7:** Dynamic response of the TF-probe under a longitudinal force.

Force (mN)	Resonance Frequency (Hz)	Probe Tip Oscillation Amplitude ( nm)	Phase Angle (°)
1E−13	32,375	10.11	91.11
0.001	32,375	10.11	91.11
0.01	32,375	10.11	91.14
0.1	32,375	10.11	91.36
1	32,375	10.09	93.59
10	32,376	9.21	114.34
20	32,377	7.57	131.51
30	32,378	6.11	142.79
50	32,380	4.21	155.36

**Figure 16:**
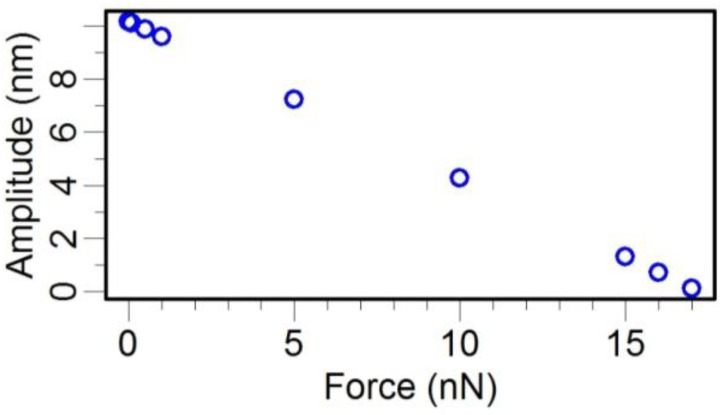
The effect of the transverse viscous force on the oscillation amplitude of the probe tip.

Second, we calculated the dynamic response of the TF-probe caused by a transverse interaction, specifically a viscous force (Figure 15b). In one vibration cycle, the maximum viscous force has the form:

(3) $$F_{viscous}=cv$$

where *v* is the maximum velocity of the probe tip, and *c* is the drag force coefficient. We applied this type of viscous force to the probe tip and changed its value from 0 nN to 17 nN. The results from a harmonic response analysis are shown in Figure 16. There was a linear relationship between the interaction force and the dynamic response of the TF-probe. The transverse force range was limited by the initial excitation force. Here, the initial excitation force incented a 10.181 nm oscillation amplitude of the probe tip, and requires a transverse force of 17 nN to decay the oscillation amplitude to 0 nm. From the point of view of force sensing, the TF-probe with 10.181 nm oscillation amplitude can detect a force less than 17 nN. In fact, the transverse force resolution was determined by measuring the oscillation amplitude. When using the TF-probe as a force sensor, if the oscillation amplitude resolution is 0.1 nm, mostly constrained by the noise level in the lab, then the force resolution can reach 0.14 nN.

## 4. Drag Force Measurement

We designed an experiment to measure the drag force, specifically the friction force, between a silica probe and a silica sample. The TF was excited at its resonance, 31.557 kHz, with a voltage of 100 mV, and the oscillation amplitude of the probe was detected by a LDV in real time. Using single degree linear vibration theory to describe this system, we can establish the following relationship:

(4) $$F_{ex}=R\Omega \gamma +F_{drag}$$

where the structure is vibrating in its resonant state and the drag force dissipates the kinetic energy of the TF-probe. Here, *F_ex_* is the effective excitation force on the TF controlled by the input voltage and its value was kept constant during the whole interaction process. *γ* is the effective damping coefficient of the TF, which includes two parts: the structure damping and air damping, and *F_drag_* is the drag force experienced by the TF, which is the friction force between the probe tip and the silicon oxide sample. 

According to linear vibration theory [45], γ can be calculated by:

(5) $$\gamma =\frac{k_{stat}}{\sqrt{3}Q\Omega }$$

where Ω is the angular frequency at resonance 198,278 rad/s, *Q* factor is 6013, *k_stat_* is the static spring constant [45], and the damping coefficient is equal to 1.4 × 10^−4^ kg·rad/s.

Before the TF interacts with the sample, there is no drag force. Thus, Equation (4) becomes:

(6) $$F_{ex}=R^{0}\Omega \gamma$$

where *R*^0^ is the effective oscillation amplitude at the free state and *R*^0^ = 190 nm in our experiment.

The friction force in our experiment can be transformed into an equivalent drag force with a linear relationship to the velocity. The usual method for this is setting the damping energy in a vibration cycle dissipated by friction equal to that of the equivalent drag force. The damping energy in one circle is:

(7) $$E_{one-circle}=4RF_{friction}=\pi RF_{drag}$$

where *F_friction_* is the friction force. Based on this, we get the equivalent drag force:

(8) $$F_{drag}=\frac{4F_{friction}}{\pi }=\frac{4\mu N}{\pi }$$

where *N* is the normal pressure between the sample and the TF, and μ is the friction coefficient. 

By combining Equations (4) and (8), we get:

(9) $$N=\frac{\pi \gamma \Omega \left(R^{0}-R\right)}{4\mu }$$

**Figure 17:**
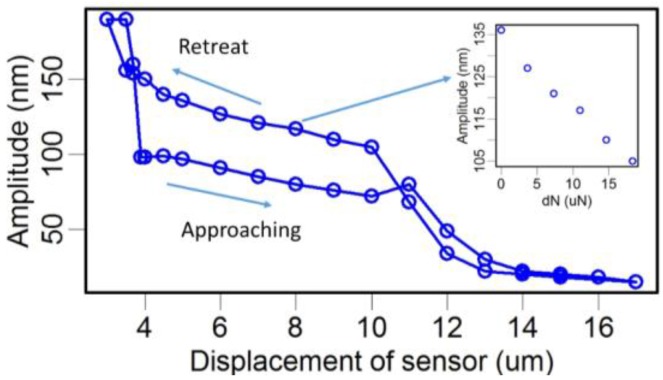
The frictional interaction between the silica–silica surfaces with varying normal pressures.

In this experiment, we recorded the whole approaching and retreat circles (Figure 17). The displacement of the sensor by the sample was linearly dependent on the normal pressure *N*. We chose the zoomed area shown in Figure 17 as the stable interaction between the TF and the sample. These results confirm that a linear relationship was observed, just as implied by Equation (9), and *dN* can be written by:

(10) $$dN=N_{i}-N_{0}$$

where *N_i_* is the normal pressure measured in the *i*th point in the zoomed area. By substituting the data in the zoomed area into Equation (9), five estimates of the friction coefficient μ were obtained that had a mean value of 0.0655. For comparison, a reference value of the sliding friction coefficient of glass-glass is 0.09–0.12 at the macroscale [47].

## 5. Discussion

### 5.1. Parameters Affecting the Resonance Frequency of the TF-Probe

The TF-probe usually works in its first or second symmetric vibration mode, in which the resonant angular frequency is:

(11) $$\Omega =\sqrt{\frac{K}{M}}$$

**Table 8:** Resonance frequency with varying probe lengths.

Probe Length (mm)	Resonance Frequency (Hz)
0.5	32,426
1.0	32,102
1.5	31,598
2.0	30,830

**Figure 18:**
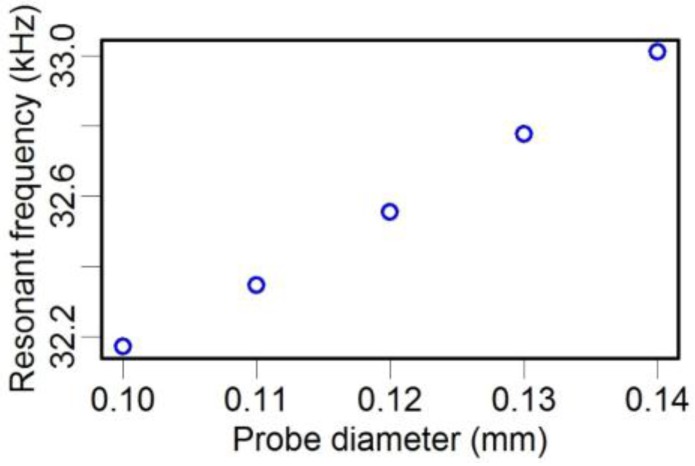
Resonance frequency with varying probe diameters.

As mentioned earlier, both stiffness and mass are related to the geometric and material parameters in a practical structure. Here, two geometric parameters have been studied, the diameter and length of the probe. We found that the resonance frequency was approximately linearly dependent on the probe diameter (Figure 18). Meanwhile, the probe length could also significantly modify the resonance frequency (Table 8). For example, when the length was varied from 0.5 mm to 2 mm, the corresponding resonance frequency of the TF-probe dropped from 32.426 kHz to 30.830 kHz. We also calculated the resonance frequency in the case of various material parameters, and these results are given in Table 9. The resonance frequency of the TF-probe increased incrementally as the Young’s modulus for both the probe and the epoxy resin were increased.

**Table 9:** Resonance frequency with Young’s modulus.

Young’s Modulus of the Probe (GPa) *	Resonance Frequency (Hz)	Young’s Modulus of the Epoxy Resin (GPa) ^#^	Resonance Frequency (Hz)
411	32,200	20	32,214
200	32,192	10	32,200
78	32,113	6	32,186
10	32,056	2	32,175

* Young’s modulus of the epoxy resin = 10 GPa; ^#^ Young’s modulus of the probe = 411 GPa.

### 5.2. Parameters Affecting the Spatial and the Force Resolutions of the TF-Probe

When a TF-probe is used in SNOM, there are two critical optimization indexes, longitudinal position control and lateral spatial resolution. The former requires the TF-probe to be very sensitive to the interaction between the probe tip and the sample surface. Besides improving the *Q* factor, enlarging the geometric size of the probe tip increased its interaction with the sample and, thus, increased the force range (Table 10). Force range is defined as the distance where the oscillation amplitude of TF-probe decreased to 95% of its initial value. The results also illustrated that changing the length of the probe tip amplified the force range effectively, which means that reducing the stiffness of the TF-probe will increase the sensitivity with which it can sense forces.

**Table 10:** Force sensitivity with the probe radii, probe length, and *Q* factor.

Probe Radii (nm)	Force Range (nm)	Probe Length (mm)	Force Range (nm)	Q Factor	Force Range (nm)
100	0.4	0.5	6.0	100	2.0
500	1.3	1.0	7.0	500	4.0
1000	2.0	1.5	10.5	3000	7.0
5025	6.0	-	-	-	-

The constraint factors of spatial resolution include the diameter of the probe tip, the distance between the probe tip and the sample surface, and the oscillation amplitude of the probe tip. For a commercial scanning near-field optical microscope, the oscillation amplitude of the TF is measureable by catching the electrical feedback of the control unit, while the oscillation amplitude of the probe tip is difficult to be detected. Therefore, artifacts will exist if the oscillation amplitude difference between the probe tip and the TF prong is neglected. Table 11 shows the effects induced by different probe lengths. When the length was below 1 mm, the ratio between the probe tip and the TF prong almost equaled 1. However, when the length range was from 1 mm to 2 mm, the ratio reached 4.92. This means that if the measured oscillation amplitude of the TF prong is 1 nm, the practical oscillation amplitude of the probe tip will be 4.92 nm, and this seriously affects the spatial resolution of the scanning near-field optical microscope.

**Table 11:** Impact of the probe length on spatial resolution.

Probe Length (mm)	Oscillation Amplitude Ratio
0.5	1.2
1.0	1.5
1.5	3.8
2.0	4.9

### 5.3. Comparison between FEM and Beam Theory

**Figure 19:**
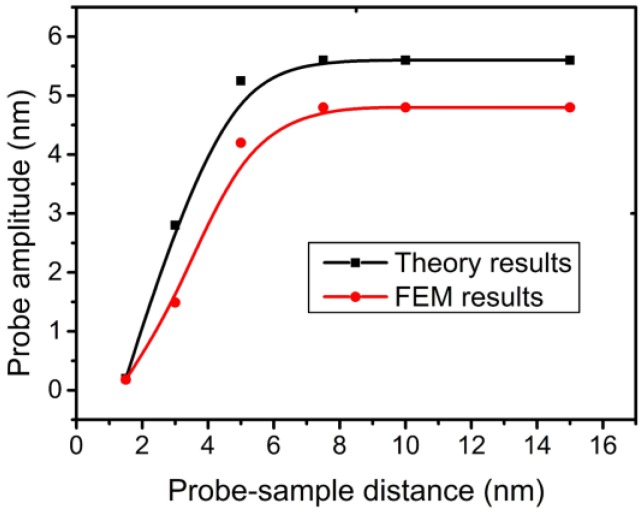
Approach curves calculated by FEM and beam theory.

The dynamic property of the TF-probe has been studied by many researchers using linear vibration theory. The control equation has the form:

(12) $$M\ddot{q}\left(t\right)+Kq\left(t\right)=\gamma {\dot{q}}_{i}\left(t\right)+F\left(t\right)$$

where *F*(*t*) is the excitation force and *q*(*t*) is the lateral modal response. The vibration mode of a TF-probe can be obtained by solving the eigenvalue equation with proper boundary conditions, and then the parameters *M*, *K*, *η*, and *F*(*t*) can be calculated using the known vibration mode and other needed parameters, including the geometric dimensions, physical property, and dynamic parameters. Generally, Equation (12) is adopted to model one prong of the TF-probe, and Figure 19 shows a comparison of the approaching curves under the Van der Waals force by using the FEM calculation and linear vibration theory. The calculation conditions are the same for these two methods. The results show that the response calculated by FEM is relatively small. The FEM calculation is more precise, because a detailed model is used (Figure 13), including the electrode pad and chrome plating, and meanwhile some computing data are confirmed with the experimental results. The linear vibration theory uses a relatively rough model. Another reason for the discrepancy in Figure 19 is that the calculation with the linear vibration theory neglects the vibration of the support area of the TF, which will consume a considerable part of the energy imported from the external excitation.

## 6. Conclusions

In this study, we made detailed measurements of the geometric dimensions and dynamic response of the TF-probe. The experimental results from changing the atmosphere pressure suggest that decreasing pressure should be a preferred way to increase the *Q* factor. Based on the measured parameters, many unknown dynamic parameters were estimated by a numerical calculation and harmonic response analysis. FEM modeling results provided a series of optimal parameters for increasing the *Q* factor, including the geometric and material parameters of the TF-probe. Various TF-probe attaching forms had a significant impact on the *Q* factor. The longitudinal and transverse interactions were investigated using the established FEM model, and the force measurement range and resolution were obtained in our calculation. A dynamic response signal measurement system based on a high-resolution LDV was established, and the friction coefficient of a silica–silica surface was obtained experimentally. Finally, the resonance frequency and spatial and force resolutions were discussed, and the comparison with beam theory illustrated the validity of using a FEM analysis to study the dynamic properties of the TF-probe.
